# Hippocampal Injections of Oligomeric Amyloid β-peptide (1–42) Induce Selective Working Memory Deficits and Long-lasting Alterations of ERK Signaling Pathway

**DOI:** 10.3389/fnagi.2015.00245

**Published:** 2016-01-11

**Authors:** Pierre Faucher, Nicole Mons, Jacques Micheau, Caroline Louis, Daniel J. Beracochea

**Affiliations:** ^1^Institut de Neurosciences Cognitives et Intégratives d’Aquitaine, Université de Bordeaux, CNRS, UMR 5287Pessac, France; ^2^Institut de Recherches ServierCroissy sur Seine, France

**Keywords:** Alzheimer disease, Aβo_(1–42)_, hippocampus, ERK/MAPK, working memory

## Abstract

Increasing evidence suggests that abnormal brain accumulation of soluble rather than aggregated amyloid-β_1–42_ oligomers (Aβo_(1–42)_) plays a causal role in Alzheimer’s disease (AD). However, as yet, animal’s models of AD based on oligomeric amyloid-β_1–42_ injections in the brain have not investigated their long-lasting impacts on molecular and cognitive functions. In addition, the injections have been most often performed in ventricles, but not in the hippocampus, in spite of the fact that the hippocampus is importantly involved in memory processes and is strongly and precociously affected during the early stages of AD. Thus, in the present study, we investigated the long-lasting impacts of intra-hippocampal injections of oligomeric forms of Aβo_(1–42)_ on working and spatial memory and on the related activation of ERK1/2. Indeed, the extracellular signal-regulated kinase (ERK) which is involved in memory function had been found to be activated by amyloid peptides. We found that repeated bilateral injections (1injection/day over 4 successive days) of oligomeric forms of Aβo_(1–42)_ into the dorsal hippocampus lead to long-lasting impairments in two working memory tasks, these deficits being observed 7 days after the last injection, while spatial memory remained unaffected. Moreover, the working memory deficits were correlated with sustained impairments of ERK1/2 activation in the medial prefrontal cortex (mPFC) and the septum, two brain areas tightly connected with the hippocampus and involved in working memory. Thus, our study is first to evidence that sub-chronic injections of oligomeric forms of Aβo_(1–42)_ into the dorsal hippocampus produces the main sign of cognitive impairments corresponding to the early stages of AD, via long-lasting alterations of an ERK/MAPK pathway in an interconnected brain networks.

## Introduction

Alzheimer’s disease (AD) is a progressive neuropathological disorder that develops over decades, leading to neuronal loss and cognitive deficits. Memory dysfunction is a core, defining characteristic of AD (Perry and Hodges, 2000) earliest manifestations being impairments in episodic memory and in working memory processing (Stopford et al., 2012). Interestingly, these two forms of memory are sustained by two main cerebral structures, namely the hippocampus (HPC) and the medial prefrontal cortex (mPFC), which are early affected in the time-course development of AD (Morris and Baddeley, 1988; Bondi et al., 2008; Webster et al., 2014). In addition, long-term memory seems to be impaired in more advanced stages of the disease (Tarawneh and Holtzman, 2012). However, the histopathological causes subtending these gradual memory deficits are still subject to controversy. Indeed, while the impact of hyperphosphorylated forms of tau proteins on memory deficits (Mandelkow et al., 2003) was investigated across the Braak stages (Braak and Braak, 1991), the causal role of the development of β-amyloid peptide (Aβ) deposits was also scrutinized as an etiological factor of AD (Hardy and Selkoe, 2002; Thal et al., 2002; Walsh et al., 2002; Shankar et al., 2008). While determining which of these biomarkers is the most representative of the disease is still under debate, recent evidence suggests a strong interplay between Aβ and tau, with tau acting as a downstream target of Aβ (reviewed in: Nisbet et al., 2015).

In the human brain, there are two main forms of Aβ species (Aβ_(1–40)_ and Aβ_(1–42)_) resulting from the proteolysis of the amyloid precursor protein (APP; Barthet et al., 2012). Under normal physiological conditions, the Aβ_(1–40)_ isoform is the most common isoform contrary to the AD pathology in which the Aβ_(1–42)_ isoform is the most abundant (Kuo et al., 1996), having the highest propensity for aggregation and leading to their accumulation in senile plaques (Andreasen and Zetterberg, 2008; Lista et al., 2014). However, findings from some brain patients suffering from AD revealed that amyloid plaques do not necessarily precede the occurrence of cognitive declines (Terry et al., 1991; Andreasen and Blennow, 2005). In contrast, important amyloid deposits have been observed in cognitively normal people (Nordberg, 2008; Villemagne et al., 2008). These observations indicate that senile plaques may not always clinically correlate with AD. Given that, it has been suggested that small soluble oligomeric species of Aβ (Aβo_(1–42)_), rather than amyloid plaques, may be neurotoxic and responsible for synaptic and network dysfunctions leading to the physiopathologic consequences of AD (Cleary et al., 2005; Haass and Selkoe, 2007; Benilova et al., 2012). Therefore, the binding of soluble Aβ assemblies to cellular membranes might trigger multiple effects affecting diverse pathways leading gradually to the cognitive manifestations of the clinical disorder (Mucke and Selkoe, 2012). To further investigate the synaptotoxicity of small isomers, experimental approaches have looked at the behavioral consequences induced by intracerebral injections of Aβ. Unfortunately, contradictory results have emerged which appear to depend on the site of injection and the nature of the peptide (for review: Chambon et al., 2011). To our knowledge, only a few studies have evaluated the impact of direct infusion of Aβo_(1–42)_ into the dorsal hippocampus on memory processes. However, recent findings showed that intra-hippocampal infusion of Aβo_(1–42)_ affected working memory when memory performance were evaluated only 10 min after the injection, suggesting that such deficits could be attributable to the acute neurotoxic effect of the peptide (Pearson-Leary and McNay, 2012). In contrast, the long-lasting effects of oligomeric assemblies of intra-HPC Aβ on working memory, which is strongly affected in early stages of AD, remain poorly characterized.

Although multiple mechanisms for Aβ peptide-induced molecular alterations and consequently memory deficits have been hypothesized there is considerable evidence implicating a role for Aβ-induced disruption of kinases critical for memory (Zhu et al., 2002; Echeverria et al., 2004; Ma et al., 2007). Among these kinases, the extracellular signal-regulated kinase (ERK) has often been claimed to be particularly sensitive to Aβ species during the course of the disease (Ma et al., 2007; Kim and Choi, 2010). However, the direction of ERK regulation by Aβ is far to end up with a clear consensus, all sorts of ERK responses to Aβ being reported (Stanciu et al., 2000; Dineley et al., 2001; Savage et al., 2002; Townsend et al., 2007; Frasca et al., 2008). On the other hand, ERK regulation in AD appears to be stage-dependent with initial activation at early stages followed by loss of activation in advanced AD (Webster et al., 2006). Beside Aβ modulation of ERK activity, ERK signaling pathway has been shown to reciprocally regulate APP metabolism and Aβ production (Cho et al., 2007). As mentioned above, we have also focused on ERK because a wide variety of studies indicate its critical importance in synaptic plasticity and memory formation (Adams and Sweatt, 2002; Nagai et al., 2006). Therefore, the alteration of the dynamic of ERK activation may alter memory processes and play a cardinal role in the genesis of memory deficits in AD.

Altogether and in view of the scarcity of the experiments exploring the potential long-lasting effect of soluble Aβ, the aim of this report was to investigate the long-term impact of Aβo_(1–42)_ injections into the hippocampus on mnemonic and molecular processes that are sensitive to AD pathology.

In the present series of experiments, we analyzed the consequences of Aβo_(1–42)_ injections into the CA1 of the dorsal hippocampus (dCA1) on two working memory tasks and on a long-term spatial memory, known to be partly sustained by this brain area. We then examined the effects of intra-dCA1 Aβo_(1–42)_ injections on memory-induced ERK1/2 activation in a functionally related brain network to better understand how local changes into the dCA1 could affect a brain circuitry during memory processing.

## Materials and Methods

### Animals

The subjects were 10-month-old naïve male mice of the C56BL/6 inbred stain obtained from Janvier (St Berthevin, France). Mice were housed individually with free access to food and water on a 12 h light-dark cycle (7:00 AM/7:00 PM) in a temperature-controlled and ventilated room. All animals were handled during 5 consecutive days before testing which were conducted during the light phase of the cycle. All experiments were approved by the local Ethics Committee for Animal Experiments and were performed in accordance with the European Communities Council Directive of 1st February 2013 (2010/63/UE).

### Surgery

Mice were anesthetized with a ketamine (1 mg/Kg body weight) − xylazine (10 mg/Kg body weight) solution and placed on stereotaxic frame. Two stainless-steel guide cannulae (26 G, 8 mm length) were implanted bilaterally 1 mm above the dorsal hippocampus according to the following stereotaxic coordinates referenced in mm from the bregma (Paxinos and Franklin, 2001; AP = −2 mm; L = ± 1, 4 mm; *V* = −0, 9 mm). Guide cannulae were fixed in place with Ionoglass dental cement (R&S Paris, France). All operated mice were manipulated every day and were allowed to recover in their home cage during 7 days before the beginning of the experiments.

### Aβ_(1–42)_ Preparation

Aβ_(1–42)_ peptide (DAEFRHDSGYEVHHQKLVFFAEDVGSNKGAIIGLMVGGVVIA) or Aβ_(1–42)_ scramble peptide (KVKGLIDGAHIGDLVYEFMDSNSAIFREGVGAGHVHVAQVEF; rPeptide, Bogart, Georgia, USA) solution were prepared as described previously (Kuperstein et al., 2010; Broersen et al., 2011; Brouillette et al., 2012). Vials containing 0.5 mg of Aβo_(1–42)_–HFIP (1, 1, 1, 3, 3, 3-Hexafluoro-2-propanol) films were allowed to defrost at room temperature for 10 min. Aβo_(1–42)_ was then dissolved in HFIP (Sigma-Aldrich) at a concentration of 1 mg/mL. The HFIP was evaporated using a gentle steam of nitrogen gas and the peptide film was dissolved in 500 μL of DMSO (Sigma-Aldrich). DMSO was totally removed of the solution by eluting the Aβo_(1–42)_ peptide from a 5 ml HiTrap desalting column (GE Healthcare) with 50 mL of buffer (pH 7.5) containing (Tris50 mM and EDTA 1 mM). Aβo_(1–42)_ peptide was then kept at −80°C in 30 μL aliquots until use. The concentration of Aβo_(1–42)_in each sample was measured using a BCA protein assay kit (Pierce, USA).

### Electron Microscopy Analysis

The biophysical and biological properties of the Aβo_(1–42)_ have been previously characterized (Kuperstein et al., 2010; Broersen et al., 2011; Brouillette et al., 2012). In our study, the preparation was analyzed using a transmission electron microscopy (TEM). Aliquots (5 μL) of the Aβo_(1–42)_ preparation were filed on carbon-coated grids (Euromedex) for 1 min. The liquid was absorbed with Whatman paper and 5 μL of dye (2% of uranyle acetate) was applied on the solution. Then, the samples were dried at room temperature during 3 min. Images were acquired using a TEM (LaB6 JEOL JEM2100), and were recorded with a camera (4 × 4 Gatan). The samples of Aβo_(1–42)_ solution were analyzed everyday from 24 h to 7 days after preparation. Each day, the preparation was analyzed 10, 30 or 60 min after thawing.

### Aβo_(1–42)_ Injections

Mice were bilaterally injected with either 0.2 μg/ μL of Aβo_(1–42)_ peptide or Aβ_(1–42)_ scramble peptide or an equal volume of buffer solution (Vehicle) into the CA1 field of the dorsal hippocampus (dCA1). Each injection consisted of the infusion of a total 0.5 μL of solution per side at a rate of 0.1 μL/min via stainless-steel cannulae (LeGuellec, FRANCE) connected to 1 μL Hamilton syringe pump system. After injections, both cannulae were left in place for the next 5 min to prevent reflux of the solution. The injections were performed once daily for 4 consecutive days.

### Histology and Determination of Beta-Amyloid Deposits

The 10 month-old mice received four infusions of Aβo_(1–42)_ into the dCA1 and were sacrificed 24 h or 7 days after the last injection. Then the presence of Aβo_(1–42)_ deposits at the time of memory testing was determined by immunohistochemistry using an antibody targeting the Aβo_(1–42)_ peptide, as described below. Cresyl violet staining was performed to evaluate the potential neuronal death caused by Aβo_(1–42)_ injections.

### Behavioral Procedures

**Figure 1:**
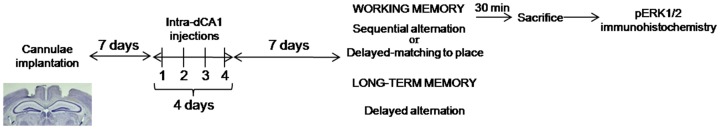
**Experimental design of behavioral experiments.** Mice received bilateral, intra-dCA1 injections of Aβo_(1–42)_ or Vehicle solution during 4 consecutive days (one injection per day). Seven days after the last injection, separate cohorts of mice belonging to the Aβo_(1–42)_ and Vehicle-infused groups were tested for working or spatial memory function using three different behavioral tasks. Working memory was tested using the sequential alternation task in a T-maze (Aβo_(1–42)_: *n* = 10, Aβ_(1–42)_ scramble: *n* = 10 and Vehicle: *n* = 18) and the delayed matching to place paradigm in the Morris water maze (Aβo_(1–42)_: *n* = 8 and Vehicle: *n* = 9); Spatial memory was evaluated using the delayed alternation task in the T-maze (Aβo_(1–42)_: *n* = 13 and Vehicle: *n* = 7). Cohorts of mice tested in the sequential alternation task were killed 30 min after the beginning of the test session for pERK1/2 immunohistochemistry.

All behavioral experiments were performed on independent cohort of animals. The general design for these experiments is depicted in Figure 1.

#### Working Memory Tasks

In a first procedure, working memory was tested in a sequential alternation task in a T-maze (Beracochea et al., 1987; Vandesquille et al., 2013; Dominguez et al., 2014). In a second procedure, working memory was evaluated in a Morris water maze using a modified version of the delayed matching-to-place paradigm (Steele and Morris, 1999).

## Sequential Alternation In a T-maze

Mice were trained on a sequential alternation task over a series of consecutive trials run in T-maze. Indeed, to alternate over a series from trial to trial, animals have both to remember at a given N trial the choice made at the N-1 trial, and to reset interfering information (trials N-2, N-3, …) which is one of the main aspect of working memory (Kesner, 2005). This task was chosen because the alternation behavior in mice is an innate tendency which does not require the use of any positive or negative reinforcement to emerge.

### Apparatus

Sequential alternation was conducted in a T-maze constructed of gray Plexiglas which is composed by a stem and two goal arms (35 × 15 × 10) and a starting box which was separated (as well as the two arms) from the stem by vertical sliding doors. The opening and closing of the doors and running latencies were automatically monitored by a computer. The apparatus was located at the center of a room with various distal cues located on the wall and a light of 30 lux.

### Training Sessions

Mice were first habituated to the T-maze and distal cues during 2 consecutive days over a 10 min free exploration session (one session per day). The next day, animals performed a training session consisting in seven successive trials separated by a 30 s inter-trial interval (ITI); during this training session, mice were familiarized with the experimental procedure (opening and closing of the doors and confinement into the goal arms). For each trial, mice remained in the start-box for a 30 s ITI. Then, the door was opened and the mouse was allowed to enter freely one of the two goal arms; the chosen arm was closed and the choice was recorded. After a 30 s confinement period into the chosen arm, the mouse was removed and brought back to the start-box for a second trial identical to the first one and this procedure was repeated over the series of trials. To avoid olfactory cues in the maze, the apparatus was cleaned using 70% (v/v) ethanol between each trial.

### Test Session

The same general procedure as that used in the training session was implemented 24 h later, except that the ITI was lengthened to 90 s. The mean alternation rate was calculated and was expressed in percentage (number of alternation/number of trials × 100). Running latencies over the series of seven trials were expressed as a mean ± SEM. To dissociate memory deficits from an eventual progressive loss of motivation to alternate over the series, an 8th trial was added to the series which was separated from the 7th trial by a shorter ITI (5 s). All animals failing to alternate at the 8th trial were excluded.

## Delayed Matching to Place Paradigm in the Water Maze

### Apparatus

The experiment was conducted in a round tank (150 cm diameter and 55 cm height) filled with water (21°C ± 1°C) made opaque with white non toxic paint. Two platforms (13 cm diameter) made of transparent Plexiglas were submerged 1 cm below water surface. One of the platforms had a 10 cm height cue thereby indicating its location during the habituation session. Several distal cues were placed on the walls of the water maze room.

### Procedure

Working memory was tested using a modified version of the delayed matching-to-place paradigm featuring an escape platform that was relocated for each daily trial set. On the first day of experiment, animals were subjected to a habituation session consisting in reaching a cued platform in a maximum of 90 s. This session was composed by four consecutive trials in which the platform location and the starting points were changed among trials. This session was aimed at familiarizing animals with the water, the room and the platform. At the end of the habituation phase, working memory was evaluated. For that purpose, the procedure was composed of four trials per day during 8 consecutive days, in which the hidden platform location changed every day. A session was composed of four trials: a first trial (acquisition trial) in which animals had 90 s to reach the escape platform; a second trial which was separated from the first one either by a 60 s or a 30 min ITI (this ITI was alternated each day during the 8-days-procedure); the remaining 3rd and 4th trials were separated by a 60 s ITI. Overall, each animal performed four sessions with 60 s ITI and four sessions with 30 min ITI between trial 1 and trial 2. Between each trial, animals were dried and returned to their home cage under a warming light pending the next trial. The starting points were randomized and were equidistant from the platform location. Several measures were used during testing. The average of escape latency (sec ± SEM) in each trial (1–4) was determined for each condition (60 s and 30 min ITIs’), as well as the savings escape latency between trials 1 and 2 corresponding to [(Latency trial 1 – Latency trial 2)/(Latency Trial 1 + Latency trial 2)] and the swim-speed average (cm/sec). Performance was recorded by a video camera located above the maze and interfaced with a computerized tracking system (ViewPoint, FRANCE).

#### Long-term Spatial Memory

This procedure was performed in the same T-maze as previously described above. Mice were submitted to the delayed alternation task using a forced-trial procedure (Dorey et al., 2012).

## Delayed Alternation Task

Initially, mice were submitted to two habituation sessions (one session per day, 10 min) in which animals were allowed to explore the maze and to familiarize with the environment. They were then given a training session as described above for the sequential alternation task. The next day, during the acquisition phase, animals were forced to enter twice the same goal arm of the maze, the access to the other arm being blocked by the sliding door. The two forced trials were separated by a 30 s ITI. Mice were subjected 24 h later to the test phase in which they were allowed to enter freely in one of the two goal arms. The correct choice (alternation behavior) is to enter in the arm opposite to that entered the day before, during the acquisition session. Animals remained for 30 s in the chosen arm and were again returned during 30 s to the starting box for a second free choice trial. This additional trial was aimed at determining whether the intrinsic psychomotor ability and motivation of mice to alternate were not impaired by the treatments, so that any deficits observed at the previous test trial cannot be ascribed to psychomotor or motivational impairments. One week later, mice were subjected to the same experimental conditions except that the blocked arm of the acquisition phase was the opposite to that of the first session. Thus, each mouse served as its own control to avoid a place preference bias.

### Immunohistochemistry and Quantification

Animals were anesthetized with an i.p injection of pentobarbital (100 mg/kg) and perfused transcardially with ice-cold 4% paraformaldehyde in 0.1M phosphate buffer (PB). Brain were removed and post-fixed overnight in the same fixation solution. Then brains were coronally-sectioned (40 μm) on a vibratome (Leica) and kept at −20°C in a 30% ethylene glycol, 30% glycerol, 0.1M PB solution until immunohistochemistry experiments as previously described (Dagnas et al., 2013). Free-floating sections were incubated for 48 h with either rabbit monoclonal antibody anti-Aβ_(1–42)_ (1:1000; Invitrogen) or rabbit polyclonal antibody GFAP (H-50; 1:500; Santa Cruz) or rabbit polyclonal anti-phospho(Thr202/Tyr204)-ERK1/2 (1:2000; Cell Signaling). Sections were then incubated with a biotinylated goat anti-rabbit IgG secondary antibody (1:2000; Jackson immunoresearch) for 2 h and then incubated with an avidin-biotinylated horseradish peroxidase complex (ABC complex; Vectastain Elite kit, Vector Laboratories). The peroxydase reaction was visualized in a Tris 0.1 M solution containing diaminobenzidine tetrahydrochloride (DAB) and hydrogen peroxide except for the GFAP antibody for which we used a DAB Nickel peroxydase substrate kit (Vector Laboratory). Sections were mounted on gelatin-coat slides, dehydrated and coverslipped. All images were acquired using an imaging analysis system (Biocom, Visiolab 2000, V4.50). The numbers of positive nuclei/mm^2^ were quantified for pERK in the CA3 field (dCA3) and dentate gyrus (DG) of the dorsal hippocampus, in the prelimbic (PL) and infralimbic (IL) areas of the prefrontal cortex, in the dorsomedial (MS) part of the septum and in the lateral nuclei of the amygdala (LA) according to Paxinos and Franklin (2001).

### Data Analysis

Statistical analyses were performed using the Statview 5.01 software (SAS Institute). The data were analyzed using one or two-way factorial analyses of variance (ANOVA), followed when adequate, by *post hoc* tests (Fisher’s PLSD). Concerning the spontaneous alternation procedure, the comparison of performance with chance were calculated with one-sample *t*-test (with hypothesized mean = chances level = 50% for correct responses). The data were presented as the mean ± SEM and were considered to be statistically significant when *p* < 0.05.

## Experimental Designs for Behavioral Experiments

### Experiment 1

Aβo_(1–42)_ injections into the dCA1 induced working memory deficits in a sequential alternation task.

Cohorts of 10-month-old mice received intra-dCA1 injections of vehicle (*n* = 18), Aβo_(1–42)_ (*n* = 10) or Aβ_(1–42)_ scramble (*n* = 10) during 4 consecutive days. Seven days after the last injection, working memory performance were evaluated in a sequential alternation task. Mice from the vehicle and Aβo_(1–42)_ groups were sacrificed 30 min after the beginning of the test session and pERK1/2 immunohistochemistry were performed. Two additional home cage control groups injected with vehicle (*n* = 5) or Aβo_(1–42)_ (*n* = 5) were sacrificed directly from their home cage for immunohistochemistry analyses.

### Experiment 2

Aβo_(1–42)_ injections into the dCA1 induced working memory deficits in a delayed-matching to place paradigm.

Cohorts of 10-month-old mice received intra-dCA1 injections of vehicle (*n* = 9), Aβo_(1–42)_ (*n* = 8) during 4 consecutive days. Seven days after the last injection, working memory was evaluated in a delayed-matching to place paradigm in a water maze.

### Experiment 3

Long-term spatial memory was spared by Aβo_(1–42)_ injections into the dCA1.

Cohorts of 10-month-old mice received intra-dCA1 injections of vehicle (*n* = 7), Aβo_(1–42)_ (*n* = 13) during 4 consecutive days. Seven days after the last injection, long-term spatial memory performance was evaluated in a delayed alternation procedure in a T-maze.

## Results

### Histological Characteristics of the *in vivo* Model After 4 Consecutive Intra-dCA1 Injections of Aβo_(1–42)_ Peptide

#### Temporal Profile of Soluble Aβo_(1–42)_ Injected Solution

**Figure 2:**
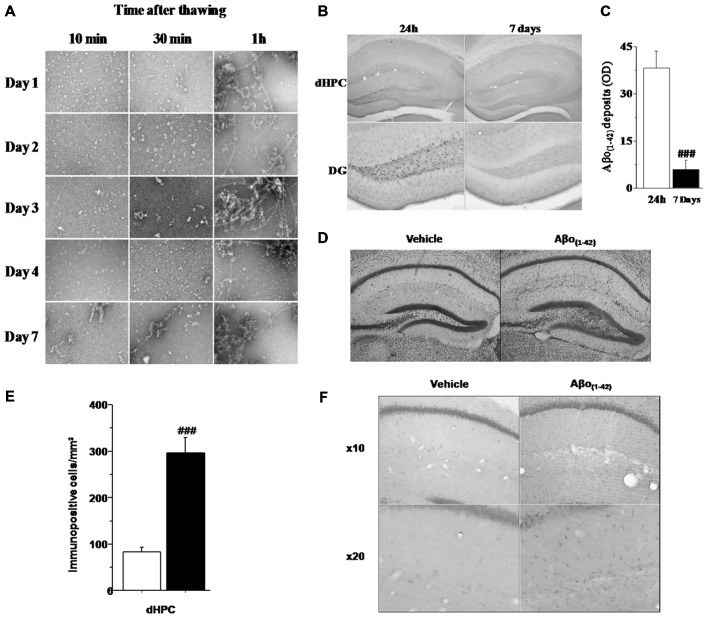
**Histological characteristics of the *in vivo* model based on four consecutive injections of Aβo_(1–42)_. (A)** Transmission electron microscopy (TEM) analysis of Aβ_(1–42)_ purified solution injected *in vivo* into the dCA1. The analysis was performed every day during 7 consecutive days at 10, 30 and 1 h after thawing. The results showed that from day 1 to day 4, the solution was mainly composed of oligomeric Aβo_(1–42)_ assemblies when thawing did not exceed 30 min. **(B)** Semi-quantitative analysis of Aβo_(1–42)_ deposits (expressed as mean optical density (OD) in the dHPC) region of mice sacrificed 24 h or 7 days after the last injection (*n* = 5 for each group). **(C)** Low magnification photomicrographs of Aβo_(1–42)_ deposits within the entire dHPC (top) and higher magnification photomicrographs of the dentate gyrus (DG; bottom) from Aβo_(1–42)_-injected mice sacrificed 24 h or 7 days after the last injection. **(D)** Representative dHPC sections 7 days after the last intradCA1 injections of Aβo_(1–42)_ or vehicle solution stained by thionine. Aβo_(1–42)_ intra-dCA1 injections induce neuronal death specifically in the DG. Effect of intra-hippocampal injections of Aβo_(1–42)_ on GFAP expression in the dorsal hippocampus. **(E)** Comparison between Aβo_(1–42)_-injected mice (*n* = 8) and Vehicle-injected mice (*n* = 8). Aβo_(1–42)_ injections into the dCA1 induce an increase of GFAP expression specifically in the dHPC. **(F)** Low magnification photomicrographs of GFAP immunopositive cells in the dHPC of Vehicle-injected and Aβo_(1–42)_-injected mice showing an increase of GFAP immunoreactivity in mice receiving intra-dCA1 injections of Aβo_(1–42)_. ^#^: *Between-group comparisons*: ^###^*p* < 0.001.

In order to evaluate the aggregation state of Aβo_(1–42)_ in our sample preparation, we assessed an analysis of the Aβo_(1–42)_ solution using TEM (Figure 2A). Examination of the amyloid peptide solution from day 1 to day 4 indicated that the preparation of soluble Aβo_(1–42)_ used was almost exclusively composed of low-molecular-weight Aβo_(1–42)_ species when samples were analyzed 10 and 30 min after thawing. In contrast, when samples were analyzed 1 h after thawing, the Aβo_(1–42)_ solution presented a high degree of aggregation. Moreover, 7 days after preparation, Aβo_(1–42)_solution exhibited almost exclusively fibrillar and aggregated species. These results led us to use Aβo_(1–42)_preparation only 4 days after preparation and more precisely, 20 min after thawing to ensure that the solution contained a large majority of low-molecular-weight species of Aβo_(1–42)_.

#### Determination of Aβ Deposits After 4 Consecutive Injections of Aβo_(1–42)_ into the dCA1

Assessment of Aβo_(1–42)_ peptide deposition in the brain of 10 month-old mice was analyzed, using an antibody which recognizes Aβo_(1–42),_ either 24 h or 7 days after the last injection using a measure of optic density (OD). As depicted by Figure 2B, the staining of Aβo_(1–42)_ deposits was restricted to the dHPC and more specifically in the DG. Otherwise, one-way ANOVA revealed that Aβo_(1–42)_ deposition was significantly reduced in mice sacrificed 7 days after the last injection as compared to 24 h-injected mice (*F*_(1,8)_ = 27.047; *p* < 0.001; Figure 2C). Consistently with the Aβo_(1–42)_ deposition in the DG, cresyl violet staining revealed a glial proliferation restricted to the DG 7 days after the last injection of Aβo_(1–42)_ (Figure 2D). However, these glial cells were located beneath the cell layer which was moderately damaged. These results suggest that intra-dCA1 injections of oligomeric assemblies of Aβo_(1–42)_ was able to induce specific and very localized neuronal damage.

#### Determination of GFAP Expression Induced by Intra dCA1 Injections of Aβo_(1–42)_

To evaluate the inflammatory effects of local infusions of Aβo_(1–42),_ we analyzed 7 days after the last injection, the immunoreactivity of GFAP in different brain structures. Figures 2E,F shows that animals receiving Aβo_(1–42)_ exhibited enhanced GFAP immunostaining which was approximately three fold higher than that found in vehicle control group (Aβo_(1–42)_ group: 296.25 ± 33.292 vs. Vehicle group: 83.625 ± 9.453). The one-way ANOVA confirmed a significant between-groups difference (*F*_(1,14)_ = 37.746; *p* < 0.001). Importantly, we found no GFAP activation in other brains structures studied (PL, SL, SM, LA; data not shown). These results indicated that Aβo_(1–42)_ injections into the dCA1 led to a specific activation of astrocytes in the dorsal hippocampus.

## Behavior

### Intra-Hippocampal Injections of Aβo_(1–42)_ Disrupt Working Memory

#### Experiment 1: Intra-dCA1 Aβo_(1–42)_ Injections Impair Working Memory Performance in the Sequential Alternation Task

**Figure 3:**
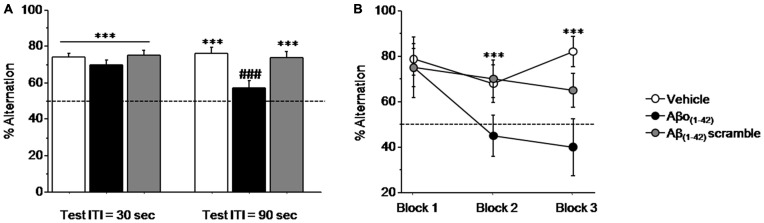
**Intra-CA1 Aβo_(1–42)_ treatment decreases working memory performance in a sequential alternation task in T-maze.** Percentage alternation performance were measured during pre-test (ITI = 30 s) and test (ITI = 90 s) sessions on Vehicle (*n* = 18), Aβo_(1–42)_ (*n* = 10) and Aβ_(1–42)_ scramble (*n* = 10) groups. **(A)** Alternation performance were impaired in the Aβo_(1–42)_ group during the test session compared with Vehicle and Aβ scramble groups. **(B)** Two trials blocks analysis indicated that alternation performance of Aβo_(1–42)_ group was impaired during successive trials. vs. 50% *chance level* ***p < 0.001; *Between-group comparisons*; ^###^*p* < 0.001.

Figure 3A (left) shows that all mice performed similarly during the training session when ITI was 30 s. Specifically, all performance expressed in percentage of alternation rates were significantly above chance level (50%) regardless of groups (Vehicle: 73.814 ± 2.278%; *t*_(13)_ = 10.452; *p* < 0.001; Aβo_(1–42)_: 70.020 ± 2.213%; *t*_(9)_ = 9.045; *p* < 0.001; Aβ_(1–42)_scramble: 75.000 ± 2.767%; *t*_(9)_ = 9.036; *p* < 0.001). One-way ANOVA confirmed no significant between-groups difference (*F*_(2,31)_ = 1.031; *p* = 0.36).

In contrast, during test session (Figure 3A right), when the ITI was increased to 90 s both Vehicle and Aβ_(1–42)_scramble groups exhibited performance significantly above chance (Vehicle: 76.179 ± 2.875%; *t*_(13)_ = 9.105; *p* < 0.001; Aβ_(1–42)_scramble: 73.350 ± 3.678%; *t*_(9)_ = 6.348; *p* < 0.001) whereas the Aβo_(1–42)_ group responded at chance (56.640 ± 4.439%; *t*_(9)_ = 1.496; *p* = 0.16). ANOVA yielded a significant effect of groups (*F*_(2,31)_ = 8.364; *p* = 0.001) and further *post hoc* comparisons confirmed that performance in the Aβo_(1–42)_ group (56.64 ± 4.439%) were significantly lower than those of Vehicle (76.179 ± 2.875%) and Aβ_(1–42)_ scramble (73.35 ± 3.678%) groups (respectively: *p* < 0.001 and *p* = 0.004), which otherwise did not differ from each other (*p* = 0.57). An additional analysis per block of two consecutive trials during the test session (Figure 3B) showed a significant between-groups difference (*F*_(2,31)_ = 11.924; *p* < 0.001) but no significant block difference (*F*_(2,62)_ = 2.010; *p* = 0.14) nor groups × blocks interaction (*F*_(4,62)_ = 1.09; *p* = 0.36). However, the Aβo_(1–42)_ group exhibited qualitatively lower alternation performance on the last two blocks of trials as compared to the Vehicle and Aβ_(1–42)_ scramble groups [Block 2: Aβo_(1–42)_: 45.000 ± 8.975%; Vehicle: 67.857 ± 8.463% and Aβ_(1–42)_scramble: 70.000 ± 8.165%] and [Block 3: Aβo_(1–42)_: 40.000 ± 12.472%; Vehicle: 82.143 ± 6.645%; Aβ_(1–42)_scramble: 65.000 ± 7.638], whereas no difference was observed on the first block [Block 1: (Aβo_(1–42)_: 75.000 ± 8.333% vs. Vehicle: 78.571 ± 6.863% and Aβ_(1–42)_scramble: 75.000 ± 13.437%].

Interestingly, all groups exhibited alternation rate significantly above chance at the 8th trial of the series, when ITI was shortened to 5 s (*p* < 0.01 in all analyses) and which did not differed between them (*p* > 0.5 in all analyses). In addition, running latencies were similar in all groups [Vehicle: 20.3 ± 0.8 s; Aβo_(1–42)_: 20.5 ± 1.6 s and Aβ_(1–42)_scramble: 23 ± 3.7 s; *F*_(2,35)_ = 1.564; *p* = 0.22].

Taken together, the whole set of results indicate that the intra-dCA1 injections of Aβo_(1–42)_ peptide but not of the Vehicle and Aβ_(1–42)_ scramble peptide led to delay-dependent working memory impairment tested in the sequential alternation task.

#### Experiment 2: Aβo_(1–42)_ Injections into the dCA1 Induced Working Memory Deficits in a Delayed-Matching to Place Paradigm

In order to confirm the results obtained with the sequential alternation task, we performed a second test based on the delayed matching to place paradigm, allowing evaluation of working memory in mice.

**Figure 4:**
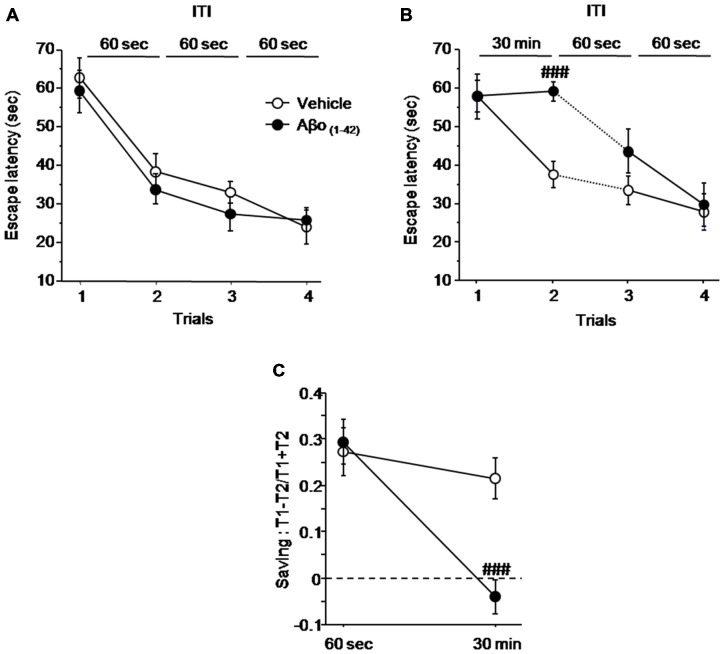
**Intra-dCA1 Aβo_(1–42)_ treatment decreases working memory performance in a delayed matching to place paradigm in the water maze.** Escape latencies (sec) as well as percentage saving were measured in Aβo_(1–42)_ (*n* = 8) and vehicle (*n* = 9) groups. **(A)** Aβo_(1–42)_ and Vehicle groups showed similar escape latency values during the four trials when the ITI was fixed at 60 s. **(B)** Aβo_(1–42)_ group exhibited a reduced escape latency value between trial 1 and trial 2 when ITI was set at 30 min, which was restored to that of vehicle group when ITI was reduced to 60 s. **(C)** Aβo_(1–42)_ group exhibited a decrease in percentage of saving when ITI was 30 min compared to vehicle group whereas no saving difference was observed when ITI was 60 s. Saving was expressed as (Latency trial 1–Latency Trial 2)/(Latency trial 1 + Latency Trial 2). *Between-group comparisons*: ^###^*p* < 0.001.

Figure 4 shows the impact of local intra-dCA1 infusions of Aβo_(1–42)_ in mice performing spatial working memory in the water maze 7 days after the last injection, compared with mice infused with Vehicle. As described in Figure 4A, all mice acquired similarly the spatial information under the ITI 60 s condition regardless of treatment. An ANOVA on mean latency to reach the platform confirmed no main effect of group (Vehicle vs. Aβo_(1–42)_: *F*_(1,15)_ = 0.629; *p* = 0.44), a significant trial effect (*F*_(3,45)_ = 33.626; *p* < 0.001) and no significant group × trial interaction (*F*_(3,45)_ = 48.215; *p* = 0.76), confirming that all mice acquired the task at the same rate. However, when the ITI between trial 1 and trial 2 was increased from 60 s to 30 min (Figure 4B), the one factor ANOVA with repeated measures yielded a significant effect of trials (*F*_(3,45)_ = 21.176; *p* < 0.001) and a significant group × trial interaction (*F*_(3,45)_ = 3.253; *p* = 0.03) but no significant effect of groups (*F*_(1,15)_ = 3.396; *p* = 0.085). The significant group × trial interaction relied on the fact that Aβo_(1–42)_ treated animals exhibited a significantly higher escape latency relative to vehicles at trial 2 (ITI = 30 min, *p* < 0.001) but not at trials 3 and 4, as the ITI was shortened to 60 s (*p* = 0.16 and *p* = 0.79 respectively). Importantly, the average swim speed was similar between Vehicle (16.494 ± 0.671 cm.s^−1^) and Aβo_(1–42)_ (15.069 ± 0.597 cm.s^−1^) groups (*p* = 0.13) indicating no effect of treatment on swim speed.

The results were further analyzed with the “saving” parameter (Figure 4C). The ANOVA analysis with repeated measures showed a significant main effect of group (*F*_(1,15)_ = 5.007; *p* = 0.04), a significant delay effect (*F*_(1,15)_ = 24.013; *p* < 0.001), as well as a significant groups × delay interaction (*F*_(1,15)_ = 11.832; *p* = 0.003). More specifically, the analysis of “saving” showed that Aβo_(1–42)_-treated group does not exhibit saving as compared to control group at the 30 min ITI (−0.4 ± 0.038 vs. 0.214 ± 0.038 respectively; *p* < 0.001). Thus, these results indicated that intra-dCA1 injections ofAβo_(1–42)_ led to delayed-dependent working memory impairment which was absent when ITI was shortened.

#### Experiment 3: Long-term Spatial Memory was Spared by Aβo_(1–42)_ Injections into the dCA1

**Figure 5:**
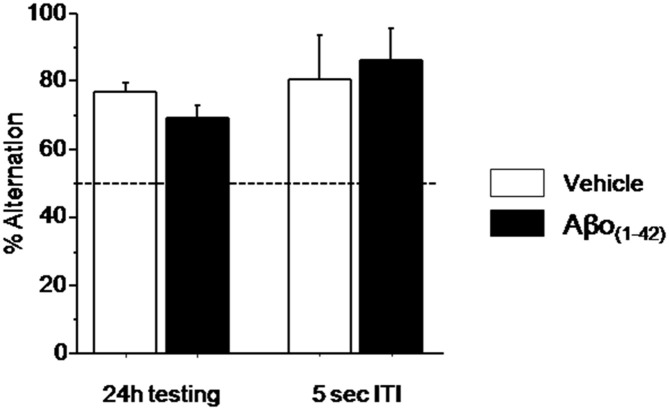
**Effects of intra-dCA1 Aβo_(1–42)_ injections on spatial delayed memory in the water maze.** Percentage of alternation was measured in Aβo_(1–42)_ (*n* = 13) and Vehicle (*n* = 7) groups at 24 h and 5 s ITI. There was significant between-group difference when animals were tested 24 h after the acquisition whereas intra-dCA1 Aβo_(1–42)_ injections did not affect spatial delayed memory at 5 s ITI.

We then examined the impact of intra-dCA1 injections of Aβo_(1–42)_ peptide on a long-term spatial memory using the delayed alternation task in the T-maze. Performance during the training session (ITI = 30 s) were significantly above chance (50%; data not shown) in Vehicle and Aβo_(1–42)_ groups (*p* < 0.01 in both comparisons) and the one-way ANOVA confirmed no significant group effect (*p* > 0.10; Figure 5).

Since the alternation performance were similar during the two test sessions, (*p* > 0.1), they were pooled for further statistical analyses. During testing session, as shown in Figure 5 alternation performance in the Vehicle and Aβo_(1–42)_ groups were significantly above chance level (Vehicle: 77.12 ± 2.335%; *t*_(9)_ = 11.614; *p* < 0.001; Aβo_(1–42)_: 69.374 ± 3.299%; *t*_(13)_ = 5.873; *p* < 0.001). Again, the ANOVA analysis performed on these data showed no significant between-group difference (*F*_(1,22)_ = 3.115; *p* = 0.09). Interestingly, both groups alternated significantly above chance level (50%) at the short-term trial [5 s of the test session (Figure 5; Vehicle: 80 ± 13.333%; *t*_(9)_ = 2.250; *p* < 0.05; Aβo_(1–42)_: 85.714 ± 9.705%; *t*_(13)_ = 3.680; *p* < 0.01)] and no significant between-groups performance was evidenced (*F*_(1,22)_ = 0.126; *p* = 0.72) indicating that the Aβo_(1–42)_ injection did not affect the motivation to alternate. These results indicated that local intra-dCA1 injections of Aβo_(1–42)_did not impact long-term spatial memory.

### Intra-Hippocampal Injections of Aβo_(1–42)_ Disrupt Patterns of Test-Induced ERK1/2 Phosphorylation in the Hippocampo-Prefrontal and Septo-Hippocampal Pathways

#### Effects of Aβo_(1–42)_ Injections on Patterns of Activated/Phosphorylated ERK1/2 (pERK) in the Dorsal Hippocampus after the Sequential Alternation Test

**Figure 6:**
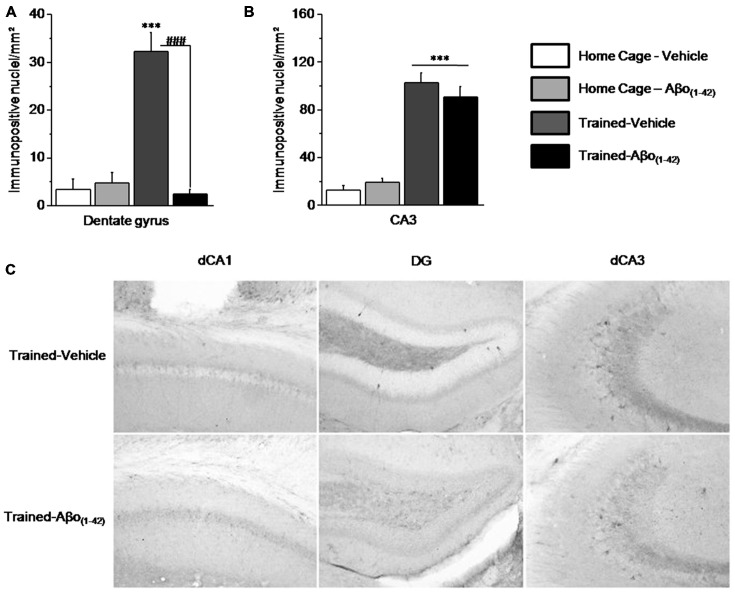
**Effects of intra-dCA1 Aβo_(1–42)_ injections on levels of phosphorylated ERK1/2 (pERK) in the dorsal hippocampus.** Tested animals were killed 30 min after the beginning of the sequential alternation test session. Levels of pERK were measured in the dorsal DG and the dorsal CA3 from Vehicle-injected mice (home-cage: *n* = 5; Tested: *n* = 10) and Aβo_(1–42)_-injected mice (home-cage: *n* = 5; Tested: *n* = 9). **(A)** Tested-related increase of pERK in the DG was abolished by Aβo_(1–42)_ injections. **(B)** In the dorsal CA3, Aβo_(1–42)_ had no effect on pERK levels in comparison with Vehicle, regardless of home-cage or tested conditions. **(C)** Representative photomicrographs of pERK immunoreactivity within the dCA1, DG and dCA3 of Trained-Vehicle (top) and Trained-Aβo_(1–42)_ (bottom) groups. ***: *Test effect*; ^###^: *Between-group comparisons*.

We compared the patterns of activated/phosphorylated ERK1/2 (pERK) in the dorsal hippocampus of Vehicle and Aβo_(1–42)_ mice sacrificed 30 min after the beginning of the sequential alternation testing session, relative to respective control Home-Cage mice. The analyses were performed on the DG and CA3 but not on the CA1 which showed no activation of ERK in each group. Two-way ANOVA evidenced a significant effect of the test (*F*_(1,25)_ = 17.558; *p* < 0.001), of Aβo_(1–42)_ (*F*_(1,25)_ = 19.735; *p* < 0.001) and a significant interaction test × Aβo_(1–42)_ (*F*_(1,25)_ = 24.089; *p* < 0.0001) in DG. As shown in Figure 6A, the interaction test × Aβo_(1–42)_ was characterized by a significant increase of pERK1/2 in Trained-Vehicle group compared to Home Cage-Vehicle group (*p* < 0.001) whereas pERK1/2 levels were similar between Trained-Aβo_(1–42)_ and matched Home Cage (*p* = 0.23). *Post hoc* analyses confirmed that Trained-Vehicle group exhibited significantly higher levels of pERK1/2 compared to Trained-Aβo_(1–42)_ (*p* < 0.0001).

In the CA3, the two-way ANOVA indicated a significant test effect (*F*_(1,24)_ = 87.348; *p* < 0.0001) but no Aβo_(1–42)_ effect (*F*_(1,24)_ = 0.092; *p* = 0.76) nor significant test × Aβo_(1–42)_ interaction (*F*_(1,24)_ = 0.981; *p* = 0.33). As depicted by the Figures 6B,C the test effect is characterized by a significant increase of pERK1/2 levels in Trained-Vehicle and Trained-Aβo_(1–42)_ compared to matched Home cage groups (both *p* < 0.0001). Moreover, no Aβo_(1–42)_ effect was observed in both Home cage and Trained groups (respectively *p* = 0.32 and *p* = 0.37).

Taken together, our data indicated that Aβo_(1–42)_ infusions into the dCA1 specifically affects levels of pERK1/2 in the DG but neither in the CA3 nor in the dCA1.

#### Distinct Patterns of pERK1/2 in Other Structures Related to the Hippocampus After the Sequential Alternation Test

**Figure 7:**
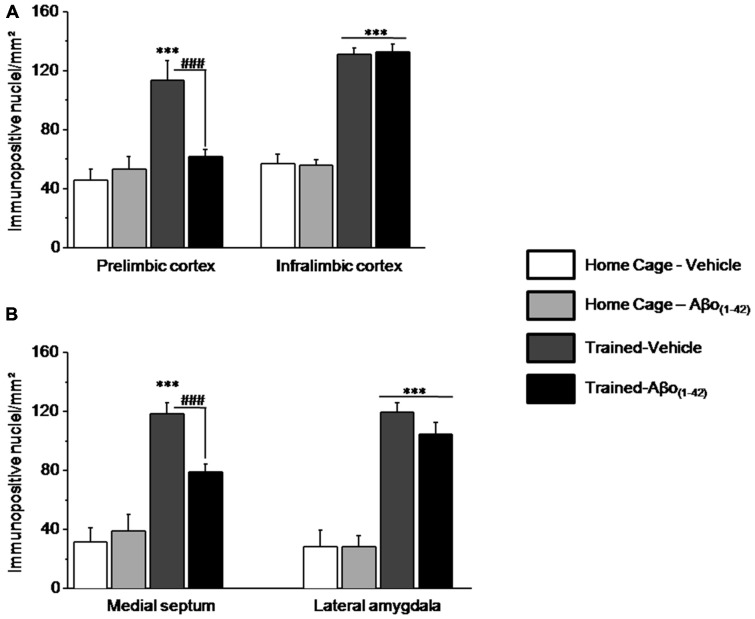
**Effects of intra-dCA1 Aβo_(1–42)_ injections on levels of pERK in other structures related to the hippocampus.** The same animals as depicted in Figure 6 were used for these analyses. **(A)** In the mPFC, levels of pERK were measured in the prelimbic (PL) and infralimbic (IL) cortices. Intra-dCA1 Aβo_(1–42)_ injections abolished tested-related increase in pERK only in the PL. **(B)** In the septum, pERK1/2 levels were inhibited by dCA1 infusions of Aβo_(1–42)_. In the lateral amygdala (LA), Aβo_(1–42)_ had no effect on pERK, regardless of home-cage or tested conditions. ***: *Test effect*; ^###^: *Between-group comparisons*.

The numbers of pERK1/2 positive cells were also quantified in the medial prefrontal cortex and more particularly in the PL and the IL cortices, in the septum (MS) and in the amygdale (LA) to evaluate whether intra-dCA1 infusions of Aβo_(1–42)_ specifically in the dHPC resulted in alterations in working memory related decrease in pERK in other structures (Figure 7). Two-way ANOVAs on pERK data from each structure revealed significant test effects [all *F* > 50; all *p* < 0.001] but no Aβo_(1–42)_ effects [PL: *F*_(1,25)_ = 4.117; *p* = 0.053; IL: *F*_(1,25)_ = 0.003; *p* = 0.95; MS: *F*_(1,25)_ = 4.01; *p* = 0.056; LA: *F*_(1,24)_ = 0.776; *p* = 0.38]. In the PL and MS, two-way ANOVAs evidenced a significant test × Aβo_(1–42)_ interaction [PL: *F*_(1,25)_ = 7.480; *p* = 0.01; MS: *F*_(1,25)_ = 8.680; *p* = 0.01] which relied on the fact that pERK1/2 increased in Trained-Vehicle compared to respective Home cage (PL: *p* = 0.003; MS: *p* < 0.0001) and that pERK1/2 levels were significantly lower in Trained-Aβo_(1–42)_ compared to Trained-Vehicle (both *p* < 0.001). In contrast, no significant test × Aβo_(1–42)_ interactions were observed in IL and LA [IL: *F*_(1,25)_ = 0.045; *p* = 0.83; LA: *F*_(1,24)_ = 0.783; *p* = 0.38]. Further *post-hoc* analyses confirmed no group difference between Trained-Aβo_(1–42)_ and Trained-Vehicle groups in these areas (both *p* > 0.5).

The whole set of results indicated that intra-dCA1 injections of Aβo_(1–42)_ led to pERK1/2 impairment specifically in the PL and the MS, while sparing the IL and the LA. These data suggest that local intra-dCA1 injections of Aβo_(1–42)_ drive changes in the PL and MS, two areas implicated in working memory.

## Discussion

We found that Aβo_(1–42)_ infusions into the dorsal hippocampus (dCA1) induced strong deficits in working memory but not in spatial memory when mice were tested 7 days after the last injection. However, the working memory deficits cannot be directly linked to specific dCA1 dysfunction. Indeed, we observed differences between the location of the primary injection site in the dCA1 and both Aβo_(1–42)_ deposits and neurodegeneration, which are located in the DG area. Even though we have no experimental explanations for this phenomenon, the accumulation of Aβo_(1–42)_ deposits in the DG could result from a mechanistic effect of the procedure used based on repeated injections, which may weaken the tissues and allowed the dispersion of the solution in the DG area. In addition, Aβo_(1–42)_ infused into the dCA1 was shown to impede the sequential alternation-induced activation of ERK1/2 in several brain structures, including the dorsal hippocampus (specifically the DG area but not the dCA1 nor the CA3), the pre-limbic cortex and the medial septum. Since the cognitive and molecular alterations were observed at a time at which amyloid plaques were no longer visible in the dorsal hippocampus, it seems reasonable to think that local infusion of Aβo_(1–42)_ has induced long-lasting molecular and cellular alterations including neuro-inflammatory processes that alter memory function.

As memory deficit constitutes the core symptom of AD, previous studies have used different animal models of AD to investigate the memory impairments that can occur during the development of the disease. The effect of Aβ peptides on different forms of memory using intracerebral injections have been contradictory and appear to depend on the site of injection, the nature of the peptide and the type of memory which was taxed (for review: Chambon et al., 2011; Webster et al., 2014). In studies with transgenic models of AD, results varied depending on age, strain and memory task. Moreover, there is no clear relationship between the appearance of amyloid plaques and working or episodic memory impairment (for review: Hall and Roberson, 2012). For example, working memory impairments in T/Y maze alternation or radial arm maze were shown before the appearance of Aβ_(1–42)_ plaques in Tg2576 mice (King et al., 1999; Arendash et al., 2004; Ohno et al., 2004), and J20 mice (Lustbader et al., 2004) while no deficit was found for PDAPP (Nilsson et al., 2004), 5 × FAD (Oakley et al., 2006; Shukla et al., 2013) or 3 × Tg mice (Carroll et al., 2007).

Numerous studies have addressed the effects of central Aβ_(1–42)_ infusion on learning and memory (Chambon et al., 2011) but no consistent results have emerged (Ghasemi et al., 2014). While some studies reported spatial memory impairment in water maze test after Aβ infusion (Majlessi et al., 2012), some others failed to show any memory deficits. Given that, some authors tried to potentiate Aβ toxicity with additional brain injuries such as ischemia (Watanabe et al., 2008), co-injection with ibotenic acid (Morimoto et al., 1998; Hruska and Dohanich, 2007) or co-injection with dexamethasone (Yao et al., 2011; Li et al., 2012). Among the few studies that have investigated the impact of local injections of Aβ into the dorsal hippocampus, most of them were infusing aggregated forms of Aβ_(1–42)_. Looking at short-term or working memory, these studies revealed impairment for spatial and non-spatial information when the animals were tested at delayed time-points after Aβ_(1–42)_ infusion (Winkler et al., 1994; Hruska and Dohanich, 2007; Christensen et al., 2008).

However, the aim of our experiments was to target an earlier stage of the disease by focusing on the oligomeric species of Aβ that have been hypothesized to be responsible for the neurotoxic effect of the peptide (Haass and Selkoe, 2007; Viola and Klein, 2015). This is why we infused small oligomeric Aβ_(1–42)_ peptide once daily for 4 consecutive days into the dorsal hippocampus and tested the animals 7 days after the last injection, a time period at which the peptides are no longer observable. Interestingly, our data clearly show no impairment of spatial memory *per se* (short- or long-term) but of working memory-related processes. As illustrated in Figure 3B, the impairment in the T-maze alternation task appears to be caused by proactive interferences as performance of experimental animals decreased dramatically only on the second block of trials. This impairment is somewhat reminiscent of previous studies from our group having shown higher sensitivity to proactive interferences in aged mice (Vandesquille et al., 2013) or mice intoxicated with chronic alcohol consumption (Beracochea et al., 1987; Dominguez et al., 2014) that might be related to hippocampal dysfunction. Although poorly investigated this higher sensitivity in proactive interference might constitute a first sign of cognitive decline in AD as it has been suggested by recent clinical data on amnesic MCI (Crocco et al., 2014).

The maintenance of information in memory over a short period of time is another aspect of working memory and this process which allows the binding of the different features of the learning situation, has been claimed to be the first to be impaired as a result of AD (Parra et al., 2009, 2010). This binding process which relies on the hippocampus (Parra et al., 2010) could be investigated in animals with the delayed matching to place task (DMTP task) in which the spatial information needs to be held in memory during a certain period of time until its use. The infusion of Aβo_(1–42)_ into the dorsal hippocampus resulted in a clear time-dependent impairment when the ITI was increased from 60 s to 30 min. Therefore, by analogy with human studies it might be speculated that platform-location binding impairment in this spatial working memory task can be specifically related to pathological changes induced by Aβo_(1–42)_ injection. Again, this might be a sensitive measure in preclinical stages of the disease (Parra et al., 2009, 2010; Pertzov et al., 2015).

The delayed spatial alternation task is showing that long-term spatial memory was not affected by the intra-hippocampal injection of Aβo_(1–42)_. This is somewhat unexpected because the hippocampus is strongly involved in the different phases of spatial memory (Morris et al., 1982; Riedel et al., 1999; McGregor et al., 2004; Martin and Clark, 2007). Parsimonious explanations would be to consider that either the Aβ toxicity is not equivalent to a lesional effect or the extent of hippocampal damages was not large enough to impede spatial learning (Moser et al., 1993). More likely, the neuropathological mechanisms induced by the Aβ infusion were too premature to alter spatial learning in tasks that are not very challenging. In support of this assumption it has been shown in Tg2576 mice that place cells degradation was observed at 16 but not at 3 months of age (Cacucci et al., 2008). No intermediary ages were studied but in the same mouse line, although synaptic plasticity and spine density were decreased in 4–5 month-old mice (Jacobsen et al., 2006) impairment in spatial learning was observed only from 6–7 months of age and accumulation of Aβ40 and Aβ42 appeared at 6–9 months (Lee and Han, 2013). Therefore, the lack of spatial memory impairment in the Aβo_(1–42)_-treated mice might correspond to a too early stage of the neuropathological alterations caused by Aβo_(1–42)_ injections.

The aim by using the immunohistochemistry with ERK1/2 was twofold: first, to analyse the Aβ influence on ERK1/2 activation and therefore, on memory formation; second, to pinpoint at a network level the consequences of hippocampal dysfunction. It is well documented that the activation of MAPK/ERK signaling cascade reflects the critical participation of different brain structures in memory formation (Dineley et al., 2002, 2007; Westerman et al., 2002; Sweatt, 2004; Trifilieff et al., 2006, 2007; Hort et al., 2007; Hoefer et al., 2008; Kim and Choi, 2010). Our results indicate that spontaneous sequential alternation induced ERK1/2 activation in all the investigated structures except in the area CA1.The lack of test-induced pERK in the CA1 could rely on the fact that (in contrast to the other structures) the delay of sacrifice did not allow detection of this marker. Nevertheless, in a parallel experiment (not reported in the present study), we showed an important test-induced activation of pCREB in the dCA1 which was blocked by Aβo_(1–42)_. However, working memory impairments caused by local intra-dCA1 injections of Aβo_(1–42)_ were associated with blockade of p-ERK1/2 immunoreactivity only in some brain structures that may constitute a functional network. The experiments were conducted 7 days after the last Aβ injection when most of the peptide has already been metabolized. This suggests that the absence of ERK1/2 activation corresponds to synaptic and plasticity detrimental effects that have been triggered by the direct impact of Aβ on cellular signaling. In indirect support of these data, soluble oligomers have been shown to directly interfere with the NMDA/ERK/CREB signaling pathway by blunting its activity in cortical neurons of Tg2576 mice and in primary hippocampal neurons (Ma et al., 2007). However, apparent conflicting reports have shown that ERK1/2 activation induced in the amygdala by cued fear conditioning was even more stimulated in APP transgenic mice (España et al., 2010). As a consequence, to better elucidate the mechanisms upstream to the delayed effects we found, it would be valuable to measure testing-induced ERK1/2 activation immediately after the last Aβ intra-hippocampal infusion.

The absence of testing-induced activation of ERK1/2 in the Aβo_(1–42)_-treated mice was observed only in a subset of the selected brain structures, namely the hippocampus with the DG, the medial septum and the pre-limbic cortex. Interestingly, these brain structures are anatomically and functionally linked (Thierry et al., 2000; Vertes et al., 2004; Spellman et al., 2015). It has been recently shown that the hippocampal-prefrontal interplay during spatial working memory encoding was mediated by gamma-frequency synchrony between the two structures (Spellman et al., 2015). In addition, the well documented septo-hippocampal axis is continuously receiving more evidence demonstrating the critical role played by the medial septum in the regulation of hippocampal spatial representation (Mamad et al., 2015). Further, ERK1/2 activation was not affected in the infra-limbic cortex and the lateral amygdala of the Aβo_(1–42)_-treated mice. These two brain structures are proposed to be part of another anatomical and functional system involved in the development of habits and in autonomic activity (Killcross and Coutureau, 2003; Vertes et al., 2004). The latter system is not supposed to be deeply involved in working memory. Altogether, our results support the view of an impairment of a specific neuronal network including the medial septum, the hippocampus and the pre-limbic cortex in Aβo_(1–42)_-injected mice, and suggest that Aβo_(1–42)_-associated working memory impairments may be related to the functional disruption of this network.

In conclusion, our results indicate that Aβo_(1–42)_ injections into the dCA1 lead to impairment of different aspects of working memory associated with ERK1/2 dysfunction in a brain circuitry involving the hippocampus, medial septum and pre-limbic cortex. Moreover, these cognitive and molecular alterations were observed 7 days after the last injection, at a time where amyloid deposits were no longer observed. These results lead us to suggest that the *in vivo* mouse model of AD based on four consecutive intra-dCA1 injections of Aβo_(1–42)_ could constitute a valuable tool to study the Aβo_(1–42)_ impact in the hippocampus which could characterize the very early stage of AD, even though in the general frame of AD, it is focused on a specific brain area which did not reflect the widespread damage occurring during the time-course of the disease. However, this model gives us new insights on the way local Aβ-induced neuropathology would spread to a neuronal network.

## Author Contributions

PF performed the behavioral and immunohistochemical experiment. CL sponsorised the study and participated to the study design and discussion of the paper, in collaboration with DJB. NM participated to the immunohistochemical study and comments on the data and JM participated to the general discussion of the manuscript.

## Conflict of Interest Statement

The authors declare that the research was conducted in the absence of any commercial or financial relationships that could be construed as a potential conflict of interest.
